# Nursing tutors’ perspectives in a vocational training course for Community Health Workers

**DOI:** 10.1590/0034-7167-2025-0056

**Published:** 2026-01-26

**Authors:** Lucas Cardoso dos Santos, Érica Gomes Pereira

**Affiliations:** I Universidade de São Paulo. São Paulo, São Paulo, Brazil

**Keywords:** Tutoring, Vocational Education, Distance Education, Community Health Workers, Professional Training., Tutoría, Educación Profesional, Educación a Distancia, Agentes Comunitarios de Salud, Capacitación Profesional.

## Abstract

**Objectives:**

to describe nursing tutors’ perspectives on the training process for Community Health Workers in a vocational course offered through distance learning.

**Methods:**

this experience report is based on the work of two nursing tutors in a free vocational course offered by a federal professional training program within and for the Brazilian Health System, between 2022 and 2023.

**Development:**

challenges related to students’ low digital literacy, limited understanding of the tutor’s role, and weak institutional support were highlighted. Asynchronous communication, heterogeneity of territories, and lack of study time compromised the training process.

**Final Considerations:**

tensions between the adopted pedagogical model and the principles of continuing education in health indicate the need to reassess vocational training policies in and for the Brazilian Health System based on an ethical-political positioning, appreciation of workers, critical digital inclusion and articulation between education and work.

## RESUMEN

**Objetivos:**

describir las perspectivas de enfermeras tutoras sobre el proceso de formación de Agentes Comunitarios de Salud en un curso técnico a distancia.

**Métodos:**

este relato de experiencia se basa en el trabajo de dos enfermeras tutoras en un curso técnico gratuito, impartido por un programa federal de formación profesional dentro y para el Sistema Único de Salud, entre 2022 y 2023.

**Desarrollo:**

se destacaron los desafíos relacionados con la baja alfabetización digital de los estudiantes, la comprensión limitada del rol del tutor y el escaso apoyo institucional. La comunicación asincrónica, la heterogeneidad territorial y la falta de tiempo de estudio comprometieron el proceso de formación.

**Consideraciones Finales:**

las tensiones entre el modelo pedagógico adoptado y los principios de la educación continua en salud indican la necesidad de reevaluar las políticas de formación técnica en y para el Sistema Único de Salud a partir de un posicionamiento ético-político, la valorización de los trabajadores, la inclusión digital crítica y la articulación entre educación y trabajo.

## INTRODUCTION

Community Health Workers (CHWs) constitute the largest Primary Health Care (PHC) workforce in Brazil, with more than 278,000 professionals linked to the Family Health Strategy^(1)^. Their work is strategic for strengthening the Brazilian Health System (In Portuguese, *Sistema Único de Saúde* - SUS), especially in the territorialized monitoring of families and communities or specific population groups. However, challenges persist in identifying health needs, understanding the social dynamics of territories, and coordinating with other team members^(2,3)^.

Although nursing regulation has advanced since its legal recognition in 2002 and the guidelines for vocational training in 2004, it is still common for many CHWs to enter practice without systematic training, which contributes to gaps in understanding their scope of practice^(4)^. This reality is aggravated by the increasing introduction of digital tools into PHC daily routine, such as cell phones and tablets, often used in an improvised manner and without proper training support^(3)^.

Since the creation of the Community Health Workers Program in the late 1980s, various training strategies have been developed, with varying focuses and responsibilities. The CHW vocational course, courses offered by the *Universidade Aberta do SUS*, and, more recently, the *Mais Saúde com Agente* Program illustrate the efforts of institutions such as vocational schools, public universities, and the Ministry of Health, often in collaboration with municipalities and health department councils. These training programs have ranged from more prescriptive models, focused on meeting programmatic goals, to pedagogical approaches that emphasize daily life problematization and collective knowledge construction, aligned with the continuing health education principles.

Information and Communication Technologies (ICTs), especially those mediated digitally, have established themselves as valuable resources for continuing education processes in health, expanding access, flexibility and personalization of learning^(5)^. However, the mismatch between current work demands and CHW training reveals the urgent need to rethink pedagogical strategies that promote the development of technical, relational and technological skills aligned with the SUS contemporary demands.

Given this scenario, and considering the 30^th^ anniversary of the Family Health Program’s creation, this experience report seeks to problematize a teaching-learning initiative aimed at training CHWs in the use of ICTs, highlighting the challenges identified throughout the tutoring process. In doing so, we aim to contribute to reflections on more powerful and contextualized pedagogical practices within the context of continuing education.

## OBJECTIVES

To describe nursing tutors’ perspectives on the CHW training process in a vocational course offered in the distance learning (DL) modality.

## METHODS

This article is an experience report on two nursing tutors’ work in a vocational training course for CHWs. Its publication is justified because nurses are recognized as a link in CHWs’ relationship and interaction with the community, acting as facilitators of teamwork in health promotion^(6)^. The authors of this report acted as nursing tutors in the course, supervising classes composed of up to 50 CHWs from different Regional Health Departments in the state of São Paulo, throughout the first edition of the program, between 2022 and 2023.

The *Mais Saúde com Agente* Program, previously called the *Saúde com Agente* Program, had its first edition offered in the 2022 and 2023 biennium. It is an innovative initiative aimed at expanding the vocational training of CHW and Endemic Disease Control Agents, the result of a partnership between the Ministry of Health, through the Department of Work Management and Health Education, the *Universidade Federal do Rio Grande do Sul* and the Brazilian National Council of Municipal Health Departments^(1)^.

The CHW vocational course, the focus of this report, aimed at practicing CHWs and developing skills for performing specific activities legally assigned to the category. It also sought to qualify professionals in the identification, prevention, and control of diseases and conditions with a view to improving healthcare work processes^(1)^.

The course was offered in a blended format, lasting ten months and with a total workload of 1,275 hours. The DL activities had a workload of 780 hours and took place in a Virtual Learning Environment (VLE), where students had access to synchronous classes, online classes, videos, films, podcasts, reading materials, educational games, discussion forums, and quizzes, always with the supervision of a tutor.

The curriculum matrix was organized into units based on three stages^(1)^:

- Introductory stage (75 hours): DL - fundamentals, VLE and tools; introduction to basic computing; language and communication; professional ethics and interpersonal relationships.
- Training stage I (780 hours): health policies, Brazilian National Primary Care Policy, Brazilian National Health Surveillance Policy in Brazil; fundamentals of health workers’ work; healthcare organization and intersectorality; geoprocessing in health, registration and territorialization; work process planning and organization; notions of epidemiology, monitoring and assessment of health indicators; health information systems, use of electronic medical records and tools to support the recording of health worker actions; work in a multidisciplinary team and intersectorality; family approach in the PHC territory; notions of microbiology and parasitology; understanding the health-disease process; knowing and building health through the environment; emerging and re-emerging diseases in the Brazilian reality; health promotion; immunization; care, education and communication in health.
- Training stage II (420 hours): environmental health; fundamentals of epidemiological, sanitary, occupational and environmental surveillance; surveillance and control of zoonoses, arboviruses and combating venomous animals; risk, vulnerability and damage to the population’s health and the environment; notions of first aid.

This report was constructed based on the systematization of reflective records prepared by nursing tutors throughout the training process, including monitoring reports, minutes of pedagogical meetings, interactions in VLE, and field notes. These records supported the analysis of perceptions, strategies, and challenges faced, allowing for the experience problematization in light of continuing health education principles.

## DEVELOPMENT

### Digital literacy in vocational training of Community Health Workers

The training experience during the CHW vocational course, within the *Mais Saúde com Agente* Program, highlighted that low digital literacy was one of the main challenges faced. Understood as the ability to consume, find, create, communicate, and share digital content in the context of healthcare work, this limitation directly impacted participants’ access to VLE and their retention in the training process.

The difficulties ranged from basic computer, tablet, and cell phone use to understanding the digital platform’s functionalities, which compromised the ability to follow activities involving video lessons, forums, quizzes, and reading materials. Added to this were recurring technical issues, such as unstable internet connections, especially in areas with poor infrastructure. Many students used their own resources, such as mobile data and personal devices, to overcome these limitations, since the devices provided by healthcare services were insufficient or nonexistent.

This reality had a direct impact on tutors’ work, who also began to provide technical support, helping CHWs with difficulties accessing VLE, resolving problems related to digital tools, and communicating with the teaching team to ensure students remained on the course.

A recent study indicates that there is still little research into the relationship between the computerization of services and the actual conditions of information records in Basic Health Units^(7)^. In the context of the course, it was observed that many CHWs accessed the content independently, without direct support from professionals with more experience using technology or from a preceptor, which increased barriers to understanding key aspects of digital training.

These limitations, however, should not be understood solely as individual failures. This is a scenario of info-exclusion^(8)^, in which unequal access to digital technologies highlights structural inequalities. Literacy must be understood beyond a technical competency, encompassing its social, economic, and political dimensions^(9)^. The lack of institutional support, the lack of public policies that guarantee minimum conditions for digital inclusion and CHWs’ historical invisibility as subjects of the training process are expressions of this scenario of exclusion.

In this regard, promoting critical digital literacy among CHWs implies recognizing that training mediated by digital technologies in the SUS also requires adequate material and institutional conditions. Digital inclusion must go beyond technical access^(8)^ and should include strengthening the autonomy, participation, and protagonism of these professionals in transforming the socioepidemiological realities in which they work^(3)^.

### Structural and pedagogical challenges in the training process

The course structure, with its asynchronous tutoring, limited the interaction between tutors and students. This, coupled with a lack of understanding of the tutors’ role as a pedagogical mediator responsible for guiding studies, clarifying doubts, and facilitating the learning process, generated a variety of feelings, including discouragement, insecurity, anxiety, and nervousness among students. These feelings required constant interaction through messaging, whether in forums or individually, to ensure better guidance and address any questions related to this issue.

Despite the course coordinator’s guidance, which established a maximum 24-hour deadline for responses to students and prohibited the use of other communication tools, such as WhatsApp^®^, asynchronous communication between tutors and students caused delays in resolving issues. This delay intensified student frustration, as they often expected immediate responses to continue with their activities, compromising the learning process fluidity.

In addition to communication difficulties, the heterogeneity of the classes also posed a significant challenge for pedagogical mediation. The differences in participant profiles regarding work reality, familiarity with digital technologies, reading and writing skills, and previous training experiences required tutors to constantly adapt their pedagogical approach. It was necessary to resize content, adjust the language, and offer individualized support, respecting learning styles and competencies.

Another limiting factor was the weak institutional support observed in some municipalities. Although the course was supported by Municipal Health Departments, many participants reported a lack of effective support from their immediate superiors and municipal management in balancing training activities with their work routines. Unlike other SUS experiences, such as the *Mais Médicos* Program, which provides for protected study hours, CHWs often had to complete course activities outside of work hours, compromising engagement and retention.

Assessing the teaching-learning process posed another challenge. Implementing relevant assessment strategies proved complex, especially given the limitations of online learning in terms of developing social and emotional skills. Practices such as plagiarism and replication of answers in different settings were also observed, requiring the adoption of differentiated and personalized approaches to prevent these behaviors and promote critical engagement among participants.

Students reported difficulties in meeting deadlines for completing and submitting assignments, attributed to the reading overload-with materials exceeding 50 pages-and the short interval between units. This situation was compounded by the expectation that practical activities, concentrated at the end of the training path, would resolve the difficulties encountered in the previous stages, which generated frustration. Added to this was the perception that certain content had little connection to professional practice and the understanding of the course as an additional work task rather than an opportunity for qualification. These factors contributed to the high dropout rates observed, distancing themselves from the logic of education through and in work.

In this context, the tutors’ role was crucial in maintaining the bond with participants and mitigating the impacts of these limitations. The ability to adapt teaching to class’s needs favored the development of CHWs’ ability to find, understand, assess, and use relevant health information^(10)^, contributing to the qualification of the work process and improvements in the care offered to the population.

### Study limitations

This experience report is based on the experiences of two nursing tutors, which limits the scope of the insights and the generalizability of the findings. Furthermore, the analysis is limited to a specific set of classes in a specific regional and temporal context, which may not reflect the full realities experienced in other regions of the country within the *Mais Saúde com Agente* Program.

### Study contributions and experience successful elements

Nursing tutors’ experience in a CHW vocational course showed that critical digital literacy, both in the DL vocational course and in PHC work, needs to be fostered with the full support of government agencies. This measure will stimulate the consolidation of digital health through the operationalization of SUS principles, aiming to promote comprehensiveness, coordination, and continuity of healthcare. Publicizing these findings also reveals the theoretical depth of the course, which can serve as a guide and trigger for planning continuing education initiatives aimed at CHWs based on the topics covered in the course.

Despite the numerous challenges faced in the tutoring context, the experience revealed important success factors from nursing tutors’ perspective, such as flexibility, active listening, a commitment to building meaningful bonds with students, and the ability to adapt pedagogically to different territorial realities and learning profiles. Despite structural limitations, tutors acted as a resource for technical and emotional support, going beyond their formal role, which contributed to the retention and engagement of many students. This expanded role reflected pedagogical care aligned with continuing health education. Furthermore, it was observed that the tutoring fostered critical reflection among CHWs on their role, the use of digital technologies, and their integration into the territory, highlighting the experience’s training potential, even in the face of adversity.

## FINAL CONSIDERATIONS

This report highlights the challenges of tutoring in a vocational training course for CHWs, revealing not only the strategic role of the tutor as a pedagogical mediator but also providing support for critical reflection on DL-mediated training processes in the healthcare field. The situations experienced offer relevant lessons for tutors’, managers’, and policymakers’ practice, in addition to paving the way for new research in the field of professional training in the SUS.

Overcoming the identified challenges requires more than technical or methodological skills; it requires sensitive communication, the ability to adapt to different territorial realities, effective institutional support, and, above all, an ethical commitment to education as a political act. It is essential that training initiatives be aligned with the principles of continuing health education, understood here as transformative praxis, focused on problematizing practices and collectively constructing knowledge with workers, not just for them.

The results of this report indicate that the obstacles faced-such as information exclusion, lack of protected study time, fragmentation between theory and practice, and weak institutional support-need to be considered in the planning and implementation of public policies for vocational training in health. For the teaching-learning process to be effective, it is necessary to train agents committed to the objective of health work: individuals’, families’, and communities’ health needs within their territories.

Hence, the implementation of professional qualification initiatives must be guided by a radical ethical commitment: the defense of emancipatory training, rooted in SUS principles, attentive to social inequalities, and committed to strengthening PHC. This allows us to envision not only the improvement of healthcare work, but also the expansion of the power of CHWs as political actors in transforming the realities in which they operate.

Finally, we recommend further studies that investigate the impacts of vocational training strategies for CHWs, especially those mediated by digital technologies. Future initiatives should consider strengthening institutional conditions to support the training process, ensuring coordination between tutors, local management, and health teams, in line with continuing health education principles.

## Data Availability

The research data are available within the article.
