# Supplementary material for: Nursing tutors’ perspectives in a vocational training course for Community Health Workers
Source: Rev Bras Enferm. 2026 Jan 26;78(6):e20250056. doi: 10.1590/0034-7167-2025-0056 (PMC12854263; doi:10.1590/0034-7167-2025-0056)
Supplement: Translation

# Translation: Perspectivas de tutores enfermeiros em um curso técnico para Agentes Comunitários de Saúde

**DOI:** 10.1590/0034-7167-2025-0056pt

**Authors:** Lucas Cardoso dos Santos, Érica Gomes Pereira

**Affiliations:** I Universidade de São Paulo. São Paulo, São Paulo, Brasil

**Keywords:** Tutoria, Educação Profissionalizante, Educação a Distância, Agentes Comunitários de Saúde, Capacitação Profissional.

## Abstract

**Objetivos:**

descrever as perspectivas de tutores enfermeiros sobre o processo formativo de Agentes Comunitários de Saúde em um curso técnico ofertado na modalidade de educação a distância.

**Métodos:**

relato de experiência, baseado na atuação de dois tutores enfermeiros em um curso técnico gratuito de um programa federal de formação profissional no e para o Sistema Único de Saúde, entre 2022 e 2023.

**Desenvolvimento:**

destacaram-se desafios relacionados à baixa literacia digital dos estudantes, compreensão limitada do papel do tutor e fragilidade no apoio institucional. A comunicação assíncrona, heterogeneidade dos territórios e ausência de tempo para estudo comprometeram o processo formativo.

**Considerações Finais:**

tensões entre o modelo pedagógico adotado e os princípios da educação permanente em saúde indicam a necessidade de reavaliar políticas de formação técnica no e para o Sistema Único de Saúde com base em um posicionamento ético-político, valorização dos trabalhadores, inclusão digital crítica e articulação entre educação e trabalho.

## INTRODUÇÃO

Os Agentes Comunitários de Saúde (ACS) constituem a maior força de trabalho da Atenção Primária à Saúde (APS) no Brasil, com mais de 278 mil profissionais vinculados à Estratégia Saúde da Família^(1)^. Sua atuação é estratégica para o fortalecimento do Sistema Único de Saúde (SUS), especialmente no acompanhamento territorializado de famílias e comunidades ou grupos populacionais específicos. No entanto, desafios persistem quanto à identificação das necessidades de saúde, à compreensão da dinâmica social dos territórios e à articulação com os demais membros da equipe^(2,3)^.

Embora a regulamentação da profissão tenha avançado desde o reconhecimento legal, em 2002, e as diretrizes para formação técnica, em 2004, ainda é comum que muitos ACS ingressem na prática sem treinamento sistematizado, o que contribui para lacunas na compreensão de seu escopo de atuação^(4)^. Essa realidade é agravada pela introdução crescente de ferramentas digitais no cotidiano da APS, como telefones celulares e *tablets*, frequentemente utilizados de maneira improvisada e sem o devido suporte formativo^(3)^.

Desde a criação do Programa de Agentes Comunitários de Saúde, no final da década de 1980, diversas estratégias de formação têm sido desenvolvidas, com enfoques e responsáveis variados. O curso técnico de ACS, os cursos ofertados pela Universidade Aberta do SUS e, mais recentemente, o Programa Mais Saúde com Agente ilustram os esforços de instituições como escolas técnicas, universidades públicas e o Ministério da Saúde, frequentemente em articulação com os municípios e os conselhos de secretários de saúde. Essas formações oscilaram entre modelos mais prescritivos, voltados ao cumprimento de metas programáticas, e abordagens pedagógicas que valorizam a problematização do cotidiano e a construção coletiva do conhecimento, alinhadas aos princípios da educação permanente em saúde.

As Tecnologias de Informação e Comunicação (TIC), em especial aquelas mediadas digitalmente, têm se consolidado como recursos valiosos para os processos de educação permanente em saúde, ampliando o acesso, a flexibilidade e a personalização da aprendizagem^(5)^. No entanto, o descompasso entre as exigências do trabalho atual e a formação dos ACS revela a necessidade urgente de repensar estratégias pedagógicas que promovam o desenvolvimento de competências técnicas, relacionais e tecnológicas alinhadas às demandas contemporâneas do SUS.

Diante desse cenário, e considerando os 30 anos da criação do Programa Saúde da Família, este relato de experiência busca problematizar uma iniciativa de ensino-aprendizagem voltada à formação de ACS no uso de TIC, destacando os desafios identificados ao longo do processo de tutoria. Ao fazê-lo, pretende-se contribuir com reflexões sobre práticas pedagógicas mais potentes e contextualizadas no âmbito da educação permanente.

## OBJETIVOS

Descrever as perspectivas de tutores enfermeiros sobre o processo formativo de ACS em um curso técnico ofertado na modalidade de educação a distância (EaD).

## MÉTODOS

O presente artigo é um relato de experiência sobre a atuação de dois tutores enfermeiros em um curso de formação técnica para ACS. Justifica-se a sua publicação porque o enfermeiro é reconhecido como elo na relação e interação do ACS com a comunidade, atuando como facilitador do trabalho em equipe na promoção da saúde^(6)^. Os autores deste relato atuaram como tutores enfermeiros no curso, acompanhando turmas compostas por até 50 ACS de diferentes Departamentos Regionais de Saúde do estado de São Paulo, ao longo da primeira edição do programa, entre 2022 e 2023.

O Programa Mais Saúde com Agente, anteriormente denominado Programa Saúde com Agente, teve sua primeira edição ofertada no biênio 2022 e 2023. Trata-se de uma iniciativa de caráter inovador voltada à expansão da formação técnica de ACS e Agentes de Combate às Endemias, fruto da parceria entre o Ministério da Saúde, por meio da Secretaria de Gestão do Trabalho e da Educação na Saúde, a Universidade Federal do Rio Grande do Sul e o Conselho Nacional de Secretarias Municipais de Saúde^(1)^.

O curso técnico de ACS, foco deste relato, teve como público-alvo os ACS em exercício profissional e como objetivo o desenvolvimento de competências para a execução das atividades específicas e atribuídas legalmente à categoria. Buscou-se, ainda, qualificar os profissionais para a identificação, prevenção e controle de doenças e agravos com vistas ao aperfeiçoamento dos processos de trabalho em saúde^(1)^.

O curso foi ofertado na modalidade semipresencial, com duração de dez meses e carga horária total de 1.275 horas. As atividades em EaD tiveram carga horária de 780 horas e ocorreram em Ambiente Virtual de Aprendizagem (AVA), no qual os estudantes tiveram acesso a aulas síncronas, teleaulas, vídeos, filmes, *podcasts*, materiais para leitura, jogos educativos, fóruns de discussão e questionários, sempre com acompanhamento de um tutor.

A matriz curricular foi organizada em unidades a partir de três etapas^(1)^:

Etapa introdutória (75 horas): EaD - fundamentos, AVA e ferramentas; introdução à informática básica; linguagem e comunicação; ética profissional e relações interpessoais.

Etapa formativa I (780 horas): políticas de saúde, Política Nacional de Atenção Básica, Política Nacional de Vigilância em Saúde no Brasil; fundamentos do trabalho do agente de saúde; organização da atenção à saúde e intersetorialidade; geoprocessamento em saúde, cadastramento e territorialização; planejamento e organização do processo de trabalho; noções de epidemiologia, monitoramento e avaliação de indicadores de saúde; sistemas de informação em saúde, uso de prontuário eletrônico e ferramentas de apoio ao registro das ações dos agentes de saúde; atuação em equipe multiprofissional e intersetorialidade; abordagem familiar no território da APS; noções de microbiologia e parasitologia; compreendendo o processo saúde-doença; conhecendo e construindo a saúde pelo ambiente; doenças emergentes e reemergentes na realidade brasileira; promoção da saúde; imunização; cuidado, educação e comunicação em saúde.

Etapa formativa II (420 horas): saúde ambiental; fundamentos das vigilâncias epidemiológicas, sanitária, saúde do trabalhador e ambiental; vigilância e controle de zoonoses, arboviroses e combate a animais peçonhentos; risco, vulnerabilidade e danos à saúde da população e ao meio ambiente; noções de primeiros-socorros.

O presente relato foi construído a partir da sistematização de registros reflexivos elaborados pelos tutores enfermeiros ao longo do processo formativo, incluindo relatórios de acompanhamento, atas de reuniões pedagógicas, interações no AVA e anotações de campo. Esses registros subsidiaram a análise das percepções, estratégias e desafios enfrentados, permitindo a problematização da experiência à luz dos princípios da educação permanente em saúde.

## DESENVOLVIMENTO

### Literacia digital na formação técnica do Agente Comunitário de Saúde

A experiência formativa vivenciada no curso técnico para ACS, no âmbito do Programa Mais Saúde com Agente, evidenciou que a baixa literacia digital foi um dos principais desafios enfrentados. Entendida como a capacidade de consumir, encontrar, criar, comunicar e partilhar conteúdos digitais no contexto do trabalho em saúde, essa limitação impactou diretamente o acesso dos participantes ao AVA e a sua permanência no processo formativo.

As dificuldades envolveram desde o uso básico de computadores, *tablets* e celulares até a compreensão das funcionalidades da plataforma digital, o que comprometeu o acompanhamento de atividades envolvendo videoaulas, fóruns, questionários e materiais de leitura. Somaram-se a isso problemas técnicos recorrentes, como instabilidade da conexão com a internet, sobretudo em territórios com infraestrutura precária. Muitos estudantes utilizaram recursos próprios, como dados móveis e equipamentos pessoais, para contornar essas limitações, uma vez que os dispositivos disponibilizados pelos serviços de saúde eram insuficientes ou inexistentes.

Essa realidade refletiu diretamente no trabalho dos tutores, que passaram a atuar também no suporte técnico, auxiliando os ACS nas dificuldades de acesso ao AVA, na resolução de problemas relacionados às ferramentas digitais e na comunicação com a equipe pedagógica para garantir a permanência dos estudantes no curso.

Estudo recente aponta que ainda são escassas as investigações sobre a relação entre a informatização dos serviços e as condições reais dos registros de informações nas Unidades Básicas de Saúde^(7)^. No contexto do curso, observou-se que muitos ACS acessavam os conteúdos de forma independente, sem apoio direto de profissionais mais experientes no uso de tecnologias ou da figura do preceptor, o que aumentou as barreiras para a compreensão de pontos-chave da formação digital.

Essas limitações, no entanto, não devem ser compreendidas apenas como falhas individuais. Trata-se de um cenário de infoexclusão^(8)^, no qual o acesso desigual às tecnologias digitais evidencia desigualdades estruturais. A literacia deve ser entendida para além de uma competência técnica, abrangendo suas dimensões sociais, econômicas e políticas^(9)^. A ausência de suporte institucional, a carência de políticas públicas que garantam condições mínimas para a inclusão digital e a invisibilização histórica dos ACS como sujeitos do processo formativo são expressões desse cenário de exclusão.

Nesse sentido, promover a literacia digital crítica entre os ACS implica reconhecer que a formação mediada por tecnologias digitais no SUS requer também condições materiais e institucionais adequadas. A infoinclusão deve ir além do acesso técnico^(8)^, devendo contemplar o fortalecimento da autonomia, da participação e do protagonismo desses profissionais na transformação das realidades socioepidemiológicas em que atuam^(3)^.

### Desafios estruturais e pedagógicos no processo formativo

A estrutura do curso com atuação assíncrona do tutor limitou os modos de interação entre tutores e cursistas que, somado ao desconhecimento acerca do papel do tutor como mediador pedagógico, responsável por orientar os estudos, esclarecer dúvidas e favorecer o processo de aprendizagem, gerou sentimentos diversos, como desânimo, insegurança, ansiedade e nervosismo nos estudantes. Esses sentimentos demandaram constante interação por meio de mensagens, seja nos fóruns ou de forma individual, para garantir melhor orientação e acolhimento das dúvidas relacionadas a essa questão.

Apesar da orientação da coordenação do curso, que estabeleceu um prazo máximo de 24 horas para as respostas aos estudantes e proibiu o uso de outras ferramentas de comunicação, como o aplicativo *WhatsApp^®^*, a comunicação assíncrona entre tutores e cursistas ocasionou atrasos na resolução dos problemas. Essa demora intensificou a frustração dos estudantes, que frequentemente esperavam respostas imediatas para seguir com as atividades, comprometendo a fluidez do processo de aprendizagem.

Além das dificuldades comunicacionais, a heterogeneidade das turmas também representou um desafio significativo para a mediação pedagógica. As diferenças nos perfis dos participantes quanto à realidade de trabalho, familiaridade com tecnologias digitais, habilidades de leitura, e escrita e experiências anteriores de formação exigiram dos tutores adaptações constantes na abordagem pedagógica. Foi necessário redimensionar conteúdos, ajustar a linguagem e oferecer suporte individualizado, respeitando estilos de aprendizagem e competências.

Outro aspecto limitador foi o frágil apoio institucional observado em alguns municípios. Embora o curso tenha contado com incentivo das Secretarias Municipais de Saúde, muitos participantes relataram ausência de suporte efetivo de suas chefias imediatas e da gestão municipal para conciliar as atividades formativas com a rotina de trabalho. Ao contrário de outras experiências no SUS, como o Programa Mais Médicos, em que há previsão de carga horária protegida para estudo, os ACS frequentemente precisavam realizar as atividades do curso fora do expediente, comprometendo o engajamento e a permanência no curso.

A avaliação do processo de ensino-aprendizagem constituiu como mais um desafio. A efetivação de estratégias avaliativas pertinentes se revelou complexa, especialmente diante das limitações do ensino *online* quanto ao desenvolvimento de habilidades sociais e emocionais. Observaram-se ainda a ocorrência de práticas como o plágio e a replicação de respostas em diferentes espaços, exigindo a adoção de abordagens diferenciadas e personalizados, com vistas à prevenção dessas condutas e à promoção do engajamento crítico dos participantes.

Os estudantes relataram dificuldades no cumprimento dos prazos para realização e entrega das atividades, justificadas pela sobrecarga de leitura - com materiais superiores a 50 páginas - e pelo curto intervalo entre as unidades. Essa condição foi agravada pela expectativa de que as atividades práticas, concentradas ao final do percurso formativo, resolveriam as dificuldades enfrentadas nas etapas anteriores, o que gerou frustração. Soma-se a isso a percepção de que determinados conteúdos apresentavam pouca relação com a prática profissional e a compreensão do curso como uma tarefa laboral adicional, e não como uma oportunidade de qualificação, aspectos que contribuíram para os índices elevados de evasão observados, distanciando-se da lógica da educação pelo e no trabalho.

Nesse contexto, a atuação dos tutores foi fundamental para sustentar o vínculo com os participantes e mitigar os impactos dessas limitações. A habilidade de adaptar o ensino às necessidades da turma favoreceu o desenvolvimento da capacidade dos ACS de encontrar, compreender, avaliar e utilizar informações relevantes em saúde^(10)^, contribuindo para a qualificação do processo de trabalho e para melhorias no cuidado ofertado à população.

### Limitações do estudo

Este relato de experiência se baseia na vivência de dois tutores enfermeiros, o que configura uma limitação no que se refere à abrangência das percepções e à generalização dos achados. Além disso, a análise está circunscrita a um conjunto específico de turmas em um determinado contexto regional e temporal, o que pode não refletir a totalidade das realidades vividas em outras regiões do país no âmbito do Programa Mais Saúde com Agente.

### Contribuições do estudo e elementos exitosos da experiência

A experiência de tutores enfermeiros em um curso técnico de ACS mostrou que a literacia digital crítica, tanto no curso técnico EaD quanto no trabalho em APS, precisa ser fomentada com apoio irrestrito dos órgãos governamentais. Tal medida estimulará a consolidação da saúde digital por meio da operacionalização dos princípios do SUS com vistas à promoção da integralidade, coordenação e continuidade do cuidado em saúde. Publicizar tais achados revela, também, a densidade teórica do curso, que pode se apresentar como norteadora e disparadora para o planejamento das ações de educação permanente voltadas aos ACS com base nas temáticas abordadas no curso.

Apesar dos inúmeros desafios enfrentados no contexto da tutoria, a experiência revelou elementos exitosos importantes do ponto de vista dos tutores enfermeiros, como flexibilidade, escuta ativa e compromisso com a construção de vínculos significativos com os estudantes e a capacidade de adaptação pedagógica diante das diferentes realidades territoriais e perfis de aprendizagem. Mesmo com limitações estruturais, os tutores atuaram como referência para o suporte técnico e emocional, extrapolando seu papel formal, o que contribuiu para a permanência e o engajamento de muitos cursistas. Essa atuação ampliada refletiu um cuidado pedagógico alinhado à educação permanente em saúde. Além disso, foi possível observar que a tutoria favoreceu o desenvolvimento de reflexões críticas entre os ACS sobre seu papel, uso de tecnologias digitais e sua inserção no território, apontando para o potencial formativo da experiência, mesmo diante das adversidades.

## CONSIDERAÇÕES FINAIS

O presente relato evidenciou os desafios no processo de tutoria em um curso de formação técnica para ACS, revelando não apenas o papel estratégico do tutor como mediador pedagógico, mas também proporcionando subsídios para a reflexão crítica dos processos formativos mediados pela EaD na área da saúde. As situações vivenciadas oferecem aprendizados relevantes para a práxis de tutores, gestores e formuladores de políticas, além de abrir caminhos para novas pesquisas no campo da formação profissional no SUS.

Superar os desafios identificados requer mais do que habilidades técnicas ou metodológicas; exige comunicação sensível, capacidade de adaptação a diferentes realidades territoriais, suporte institucional efetivo e, sobretudo, um compromisso ético com a educação como ato político. É fundamental que as ações de formação estejam alinhadas aos princípios da educação permanente em saúde, entendida aqui como práxis transformadora, voltada à problematização das práticas e à construção coletiva do conhecimento com os trabalhadores, não apenas para eles.

Os resultados deste relato apontam que os obstáculos enfrentados - como a infoexclusão, a ausência de tempo protegido para estudo, a fragmentação entre teoria e prática, e o frágil apoio institucional - precisam ser considerados no planejamento e na implementação de políticas públicas de formação técnica em saúde. Para que o processo de ensino-aprendizagem seja efetivo, é necessário formar agentes comprometidos com o objeto de trabalho da saúde: as necessidades de saúde dos indivíduos, famílias e coletividades a partir de seus territórios.

Nesse sentido, a implementação de ações de qualificação profissional deve se orientar por uma aposta ética radical: a defesa de uma formação emancipadora, enraizada nos princípios do SUS, atenta às desigualdades sociais e comprometida com o fortalecimento da APS. Assim, é possível vislumbrar não apenas a melhoria do trabalho em saúde, mas a ampliação da potência dos ACS como sujeitos políticos na transformação das realidades em que atuam.

Por fim, recomendam-se novos estudos que investiguem os impactos das estratégias de formação técnica para ACS, especialmente aquelas mediadas por tecnologias digitais e que futuras iniciativas considerem o fortalecimento das condições institucionais de apoio ao processo formativo, garantindo a articulação entre tutores, gestão local e equipes de saúde, em consonância com os princípios da educação permanente em saúde.

## Data Availability

Os dados da pesquisa estão disponíveis no corpo do artigo.
